# Characterisation of White Metakaolin-Based Geopolymers Doped with Synthetic Organic Dyes

**DOI:** 10.3390/polym14163380

**Published:** 2022-08-18

**Authors:** Antonio D’Angelo, Giovanni Dal Poggetto, Simona Piccolella, Cristina Leonelli, Michelina Catauro

**Affiliations:** 1 Department of Engineering, University of Campania “Luigi Vanvitelli”, Via Roma n. 29, 81031 Aversa, Italy; 2 Department of Environmental, Biological and Pharmaceutical Sciences and Technologies, University of Campania “Luigi Vanvitelli”, Via Vivaldi 43, 81100 Caserta, Italy; 3 Department of Engineering “Enzo Ferrari”, University of Modena and Reggio Emilia, Via P. Vivarelli 10, 41125 Modena, Italy

**Keywords:** metakaolin, synthetic organic dyes, FT-IR, CIELAB space, XRD, XRF

## Abstract

Over the years, many materials have been used to restore buildings, paintings, ceramics, and mosaic pieces exhibiting different types of dyes and colour hues. Recently, geopolymers have been used for restoration purposes owing to their high chemical and mechanical resistance. In this work, white metakaolin was used to obtain white geopolymers, cured at 25 and 40 °C, as bulk materials to be coloured with synthetic organic dyes, i.e., bromothymol blue, cresol red, phenolphthalein, and methyl orange. These dyes were added during the fresh paste preparation to obtain dyed geopolymeric solids. Ionic conductivity and pH measurement confirmed the chemical stability of the consolidated materials, while FT-IR analyses were used to follow the geopolymerisation occurrences at different ageing times (from 7 to 56 days). Finally, the colour hues and properties were assessed in the CIELAB colour space before and after immersion in water.

## 1. Introduction

It is well-known that for aesthetic purposes, coloured pigments, inorganic or organic, are added to white, and sometimes even grey, Portland cement, with contents ranging from approximately 0.1% to 14% depending upon which colour is desired [1]. As an example, potassium ferrocyanate dyes colour cement paste with a strong blue hue, while tannic acid dyes impart a dark brown colour [2]. Some of these pigments are pH-dependent and may influence the rate of chemical reaction, whilst others are effectively inert.

As alternatives to Portland cement, geopolymers, or alkali-activated binders, can be produced using different aluminosilicate powders and alkaline solutions [3]. Metakaolin (MK) is the preferred aluminosilicate powder for reference systems when structural investigations on the 3D geopolymeric structure are conducted [4,5,6]. Due to the versatility of the MK-based geopolymers, colouration has been investigated over the years, particularly using inorganic pigments [7].

Concerning the colouration with organic molecules of alkali-activated materials and/or geopolymers, a paper [8] reported the possibility of incorporating acid-base indicators (bromothymol blue, thymol blue, bromocresol green, phenolphthalein, thymolphthalein, and methyl red) in meta-halloysite-based formulations. Low concentrations of indicators (0.01–0.5 mmol) were explored to measure the colour change from dry to wet state of the solidified geopolymer material in the required pH range.

Recently, mimicking the nanostructured “Maya Blue” has been studied to synthesise coloured geopolymer by mixing sepiolite/dye (methylene blue and methyl red) with metakaolin before the addition of the potassium silicate used for geopolymerisation [1]. Moreover, a recent study reported the stabilisation of natural dyes, extracted from plants, into nanofillers such as white pozzolan, metakaolin, Lipari pumice, and silica [9]. Aiming to prepare a new kind of coloured geopolymer suitable for both design and restoration purposes, in this study, we investigated the possibility of obtaining coloured geopolymer by dissolving four different organic dyes into the fresh paste of a white metakaolin to develop the characteristic colour of the alkali media. After a complete characterisation of white geopolymers reticulated and cured at 25 or 40 °C and tested at different ageing times (from 7 to 56 days), the obtained results were compared with those of coloured geopolymers extracted after 56 days of ageing. In particular, the chemical stability was investigated by pH and ionic conductivity, whereas Fourier transform infrared spectroscopy (FT-IR) was used to follow the geopolymerisation occurrences. In addition, the ability to leach heavy metals from MK and the white geopolymers was investigated. Finally, the stability of the colour was assessed in the CIELAB colour space before and after immersion in water.

## 2. Materials and Methods

### 2.1. Materials

#### 2.1.1. Metakaolin (MK)

An ultra-white metakaolin (MK) was purchased from IMCD Deutschland GmbH & Co. (Köln, Germany). The chemical oxide composition of the white metakaolin powder (Table 1) was determined by energy-dispersive X-ray fluorescence (ED-XRF) spectroscopy with a Shimadzu Spectrometer EDX-720 (GmbH, Duisburg, Germany) equipped with a 50 W Rh target X-ray tube, a high-energy resolution Si (Li) detector, and five primary X-ray filters. The measurement was performed in the ranges of Na-Sc and Ti-U. Table 1 shows that silica, alumina, titania, and iron oxides were the main components of this raw material.

**Figure 1:**
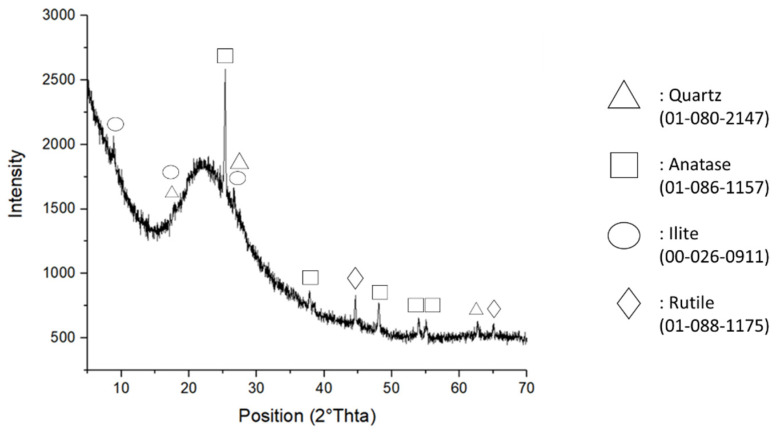
XRD pattern of the as-received white metakaolin powder.

The crystalline phases of the MK powder were identified by X-ray diffraction (XRD) attained with an X’Pert PRO, PANAlytical, (Malvern Panalytical Ltd., Malvern, UK) diffractometer operated at 40 kV and 40 mA using Cu-Kα radiation (Ni-filtered). The diffraction pattern was collected by an X’Celerator detector (Malvern Panalytical Ltd., Malvern, UK)from 5 to 70° 2θ with a step size of 0.02° 2θ and a counting time of 3 s. Mineral phases were identified by comparing the experimental peaks with reference patterns (DIFFRAC plus EVA software, 2005 PDF2, Bruker, Billerica, MA, USA). Figure 1 reports the MK XRD spectrum, which confirms that the MK was mainly amorphous with crystalline phases assigned to TiO_2_ (anatase and rutile), illite, and traces of alpha quartz.

**Figure 2:**
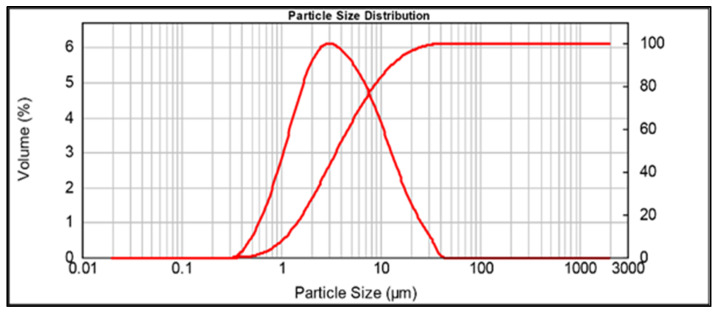
Particle size distribution of the as-received white metakaolin powder.

MK granulometry was investigated by a Mastersizer 2000 (Malvern Instruments Ltd., Malvern, UK) and is reported in Figure 2. The particle size distribution was d(01): 1.128 µm, d(05): 3.613 µm, and d(09): 12.863 µm.

#### 2.1.2. Alkaline Solutions

Sodium hydroxide, NaOH, solution containing 8 mol/L in MilliQ water and sodium silicate, Na_2_SiO_3_, solution (SiO_2_/Na_2_O = 2.58 M ratio; SiO_2_ = 26.50 wt%, Na_2_O = 10.6 wt% and pH = 12.5, Ingessil, Verona, Italy) were used as the alkaline activator for the geopolymer synthesis.

#### 2.1.3. Organic Dyes

Four pH indicators were used as dyeing molecules:

Phenolphthalein (PPT), (3,3-bis(4-hydroxyphenyl)-2-benzofuran-1(3H)-one): In an acid, it is colourless and in alkali conditions between pH 8.3 and 10.0 it is fuchsia; above that value, it starts fading until returning colourless at pH = 13 or above. MM: 318.33 g/mol.

Bromothymol blue (BB), (3,3-bis[3-bromo-4-hydroxy-2-methyl-5-(propan-2-yl)phenyl]-2,1λ6-benzoxathiole-1,1(3H)-dione). In an acid, it is yellow; in alkali conditions, it is blue. The pH range is 6.0–7.6. MM: 624.38 g/mol.

Methyl orange (MO), (sodium 4-{[4-(dimethylamino)phenyl]diazenyl}benzene-1-sulfonate). In an acid, it is reddish; in alkali conditions, it is yellow. The pH range is 3.1–4.4. MM: 327.33 g/mol.

Cresol red (CR), (3,3-Bis(4-hydroxy-3-methylphenyl)-2,1λ6-benzoxathiole-1,1(3H)-dione). It changes colour in the pH range of 7.2–8.8 from yellow to purple. MM: 382.44 g/mol.

In particular, three of the indicators were of the triphenylmethane type and one (methyl orange) was of the azo type; they are soluble in alkaline solutions and were incorporated into the geopolymer by dissolving 500 mg of the powder of each dye into the proper aliquot of the NaOH/Na_2_SiO_3_ activating solutions before geopolymer synthesis. All the reagents were purchased from Sigma-Aldrich (Darmstadt, Germany).

### 2.2. Geopolymers Synthesis: White and Coloured Samples

**Figure 3:**
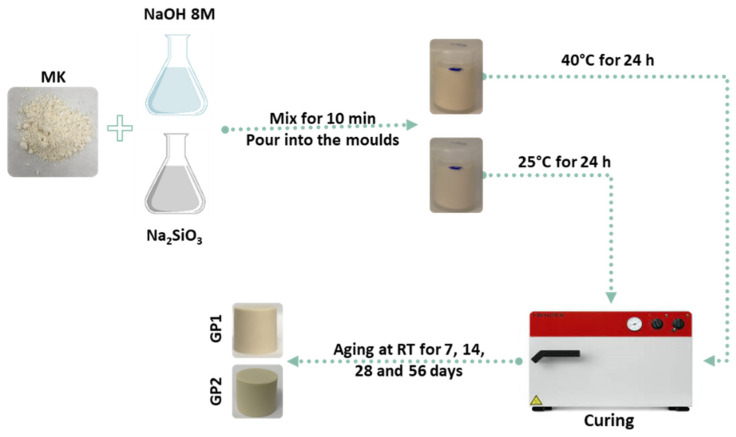
Flow diagram procedure of white geopolymer synthesis.

Figure 3 depicts a schematic flow-chart procedure of the white geopolymer synthesis. The geopolymers were produced by adding 8 M NaOH and sodium silicate solution to dried MK powder under mechanical stirring with SiO_2_/Al_2_O_3_ = 2.7 and Na_2_O/Al_2_O_3_ = 0.48 ratios, with a 32% water content, as previously reported [2]. The fresh pastes were poured into plastic moulds and cured at 25 or 40 °C for 24 h before ageing at room temperature (RT) for 7, 14, 28, and 56 days. The samples cured at 25 °C are labelled GP1, while GP2 are the ones cured at 40 °C. The dyed geopolymers were obtained following the procedure described above, but using coloured NaOH solutions. All the coloured geopolymers were characterised after an ageing time of 56 days. The coloured geopolymer samples were named according to their label and the curing treatment (for example, the geopolymer obtained with phenolphthalein and cured at 25 °C for 24 h is labelled as PPTGP1, while the one cured at 40 °C for 24 h is named as PPTGP2).

### 2.3. Geopolymer Characterisation

#### 2.3.1. Ionic Conductivity and pH

Indirect information about the geopolymer’s stability can be obtained by measuring both pH and ionic conductivity (IC) over time. To this aim, Milli-Q water (1:10 *w:v*) was added to the ground and sifted (d < 125 µm) samples. After shaking, waiting for the sedimentation, and filtering (d < 0.45 µm) under vacuum the solutions, the values of pH and ionic conductivity of the eluate were collected after 1 h [3,4]. IC measurements were performed with a Crison GLP31 (conductivity cell 50 72, made up of glass and platinum, measuring range from 0 to 50,000 µS/cm, temperature range from −35 to 85 °C), whereas pH measurements were performed with a Crison GLP21 (pH measuring range 0–14, reference element of Ag/AgCl with Ag+ ion barrier, operating temperature range −10 to 100 °C). Crison GLP21 and 31 instruments were from Hach Lange Spain, S.L.U, Barcelona, Spain.

#### 2.3.2. Integrity Tests and Colour Evaluation

The integrity of the sample after immersion in water and the consequent weight loss were used as indirect tests to investigate the stability of the geopolymeric materials [5]. The samples were broken into large pieces, dried in acetone and then in air, and finally weighed and soaked in Milli-Q water (1:100 *w:v*) for 24 h. After 24 h, observations were recorded (see Section 3.1.2), and the pieces were extracted from the water and immersed for 3 h in acetone (reagent grade, Sigma-Aldrich, Darmstadt, Germany). After air-drying for 3 h at RT, the sample was weighted.

The colour of the geopolymers was measured at the surface using a colourimeter (Colorimeter PCE-CSM 6, PCE Holding GmbH, Hamburg, Germany) in the UV–visible range of 300–800 nm. The equipment automatically defines the colour in the CIELAB space. When light passes through an object, some of it is absorbed and, as a result, there is a decrease in the amount of reflected light providing the three values: L* (lightness), a* (red to green hue), and b* (blue to yellow hue) to univocally define the colour with three numerical parameters. We adopted the ∆E to evaluate the colour difference between two different samples: n (sample with organic dye) and m (sample without organic dye), defining:

∆L* = L*n − L*m(1)

∆a* = a*n − a*m(2)

∆b* = b*n − b*m(3)

and calculating ∆E as:

(4) $$∆E=\;\sqrt{\left(∆L^{*2}+∆a^{*2}+∆b^{*2}\right)}$$

Differences in the ∆E between two samples as small as 3 to 5 units are almost imperceptible by the human eye. Values of ∆E lower than 3 are not perceptible; higher than 5, the difference in colour is very evident [6].

#### 2.3.3. FT-IR Analysis

Fourier transform infrared (FT-IR) spectroscopy analysis was performed in the range of 400–4000 cm^−^^1^ by a Prestige21 Shimadzu system (Shimadzu, Milan, Italy), equipped with a deuterated triglycine sulphate with potassium bromide windows (DTGS KBr) detector with a resolution of 2 cm^−1^ (60 scans). The analysis procedure used KBr disks (2 mg of sample mixed with 198 mg of KBr, reagent grade, Sigma-Aldrich, Darmstadt, Germany). FT-IR spectra were elaborated by IRsolution (v.1.60, Shimadzu, Milan, Italy) and Origin. (v.2022b, OriginLab Corporation, Northampton, MA, United States). The analyses were performed on the samples extracted after 7, 14, 28, and 56 days of ageing time at room temperature.

#### 2.3.4. Leaching Test

The leaching of heavy metals from MK powder and geopolymers was carried out according to EN 12457-2:2004 [10]. Samples, crushed and sieved to particle sizes less than 4 mm, were placed in bidistilled water with a 10 L/kg liquid/solid weight ratio, and maintained for 24 h. After the recovery of the filtered (d < 0.45 µm) leachate solutions, they were acidified with HNO_3_ (69%) solution to pH 2. Then, according to BS EN ISO 11885:2009 [11], ionic heavy metal concentrations were determined by ICP-OES (Agilent, Santa Clara, CA, USA). All the ionic metal concentrations are expressed as parts per million. The limit of detection (LOD) for Al, B, Ba, Fe, Mn, Sb, and Zn was 5 ppb; the LOD of Be, Cd, Co, Cr, Cu, Mo, Ni, Pb, Se, Sn, and V was 2 ppb; and the LOD of Si and Ca was 500 ppb. 

## 3. Results and Discussion

### 3.1. Characterisation of Geopolymer Samples

**Figure 4:**
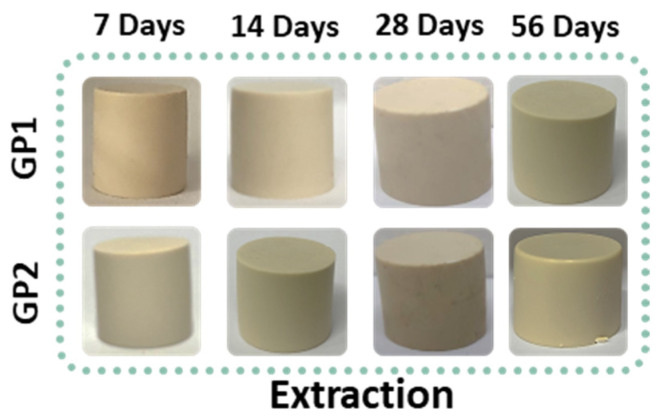
GP1 and GP2 samples extracted at 7, 14, 28, and 56 days.

Figure 4 reports the white geopolymers sample after the extraction procedure at different curing (25 and 40 °C) and ageing times at room temperature. All the samples were white/milky with smooth surfaces and resistant to finger pressure. A few bubbles were located on the upper surfaces of the samples extracted after 28 days, and in the other samples, they generally were located at the upper surface of the cylindrical shapes.

**Figure 5:**
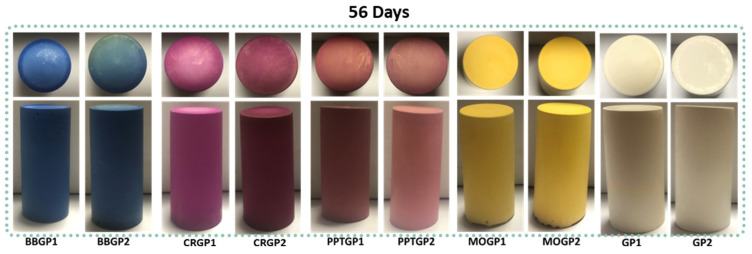
Images of coloured geopolymers after 56 days of ageing time. GP1 and GP2 are the respective controls for the sample cured for 24 h at 25 or 40 °C.

To better understand the possibility of synthesising coloured geopolymers by using the same conditions of GP1 and GP2, the dyeing pH indicator powders were directly dissolved into the NaOH/Na_2_SiO_3_ solution used for the geopolymerisation. Figure 5 reports the top and side view of the samples after 56 days of ageing. The curing at 25 and 40 °C affected the hue of the colours mainly for the geopolymer samples with phenolphthalein and cresol red. Focusing on the top view of BBGP2, we observed a little colour change from blue to green. This change could have been due to the efflorescence phenomenon that slowly led to acidifying the geopolymer surface by carbonate formations. The dyed geopolymers were also removed from the mould after one year and no differences in the aesthetical aspect were noted.

Through the colour measurement, at the two bases of each cylinder as well as on the side, the average ∆E parameter was calculated from the Equation (1) to evaluate any eventual colour difference between the GP1 and GP2 series of coloured formulations. The colours of the two series (Table 2) result very similarly, within a difference of 3 points in ΔE, except in the case of samples CRGP1 vs. CRGP2, and PPTGP1 vs. PPTGP2. In these cases, the higher temperature of GP2 with respect to GP1 and the alkaline environment have enhanced the high pH form, being for phenolphthalein the colourless one.

**Figure 6:**
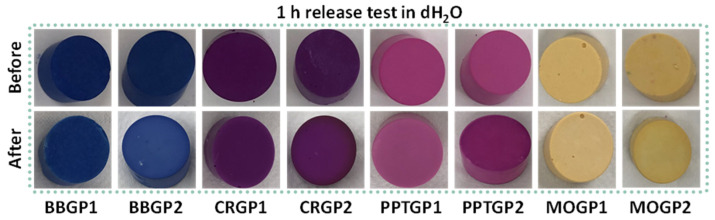
Images of coloured geopolymers before and after 1 h of release in water.

The coloured GPs were subjected to a 1 h release test to evaluate if the samples held their colours without any further treatment. After the test, the colours of all the samples lighted by 15–50 points in ΔE values, as shown in Figure 6 and Table 3.

#### 3.1.1. Ionic Conductivity and pH Measurements

**Figure 7:**
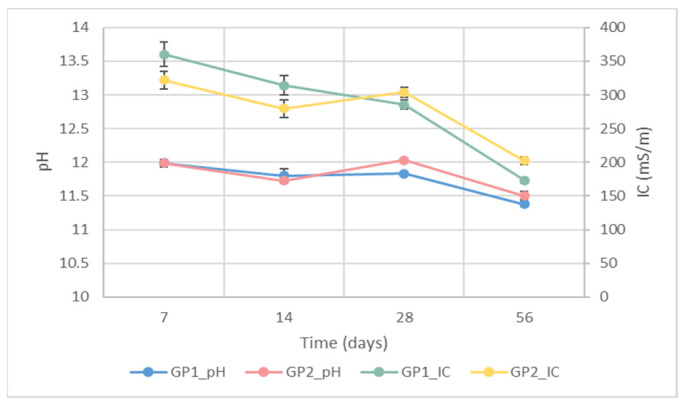
IC and pH values of the eluates obtained after samples GP1 and GP2 were immersed for 1 h in water at different ageing times.

IC and pH were evaluated to obtain indirect data about the stability of the synthesised GP1 and GP2 geopolymers cured at different times. According to [3], an uncomplete reaction between aluminosilicate powder and NaOH/Na_2_SiO_3_ solution leads to variations in both the pH and IC values of the water after sample immersion. Figure 7 shows the pH of GP1 (blue dot and line) and GP2 (red dot and line) cured for different lengths of time. The graph reveals that there were no differences between the pH values of the geopolymers cured at 25 or 40 °C. Moreover, the pH values stabilised at 11.4 and 11.5 after 56 days of ageing, suggesting that there were no large pH variations over time. Regarding the IC, for GP1 (green dot and line) and GP2 (yellow dot and line), there were two trends. GP1 showed a gradual reduction in IC values (from 360 to 175 mS/m) over time, whereas GP2 showed a decreasing trend up to 14 days of ageing, followed by a little increase (from 275 to 300 mS/m), finally settling at 200 mS/m. Finally, the graph also underlines that, after 56 days of ageing, both the pH and IC values of the samples cured at 40 °C for 24 h were slightly higher than those obtained for GP1 (cured at 25 °C).

**Figure 8:**
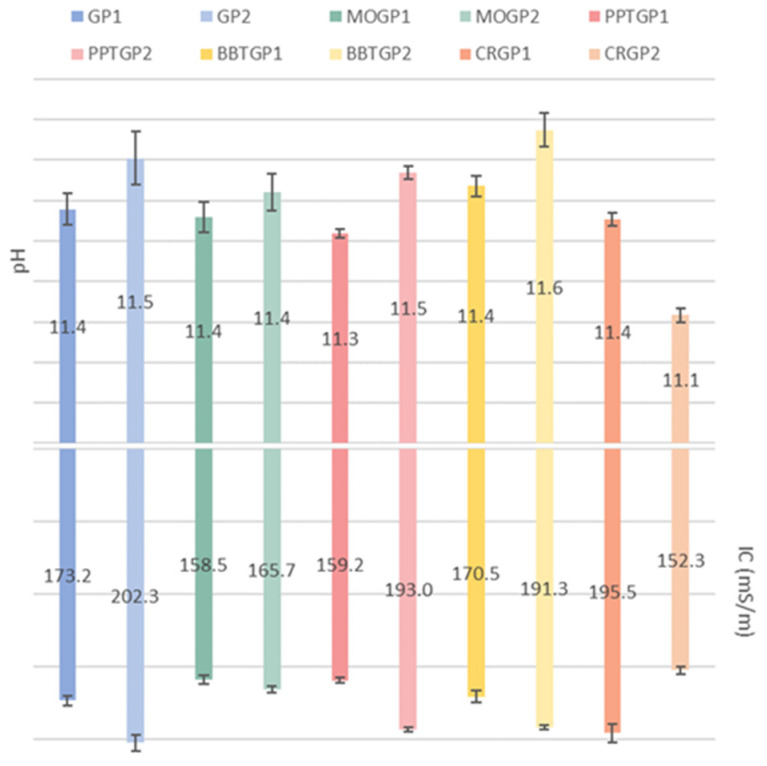
pH and IC values of the eluates obtained after 1 h immersion of the coloured geopolymer samples in water, cured at 25 or 40 °C and tested after 56 days of ageing time.

The pH and IC data of the coloured geopolymer samples (56 days of ageing time) are shown in Figure 8. The histogram reveals that the pH values of all the coloured geopolymers are similar to those obtained by GP1 and GP2 after 56 days of ageing. Only CRGP2 had a lower pH value (11.1) than all the other samples (11.3–11.6). Moreover, CRGP2 had a lower IC value than all the other coloured geopolymers. As noticed for the white GP1 and GP2 geopolymers, the differences between the curing temperatures of the dyed geopolymers also resulted in slight increases in both the pH and IC values.

#### 3.1.2. Integrity and Weight Loss Tests

All the GP samples passed the integrity tests. In Table 4, all the results of the integrity tests are summarised. The GP eluates from the integrity tests were clear and the pH measurements were alkaline, ranging from 9 to 10.4. Comparing all the results, we found no differences between the samples cured at the two temperatures.

**Figure 9:**
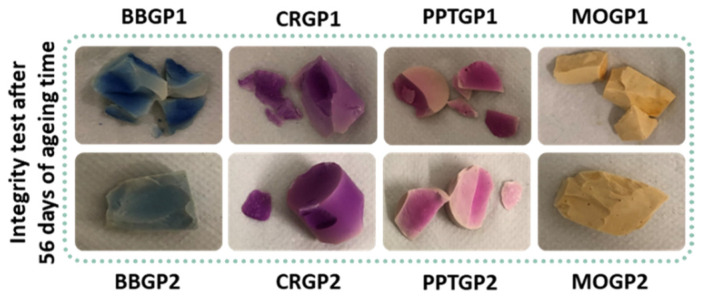
Images of the coloured geopolymers after integrity tests at 56 days of ageing.

Figure 9 shows images of the coloured geopolymers tested after 56 days of ageing. All the samples cured at 25 °C (GP1 series) did not pass the test; they were broken. The BBGP2 and MOGP2 samples were intact after the test, whereas CRGP2 lost a little piece and PPTGP2 broke like the samples of the GP1 series. The integrity test images revealed that all the coloured geopolymers partially lost their colours after 24 h of soaking in Milli-Q water. Finally, the water pH values ranged from 9.5 to 10.

**Figure 10:**
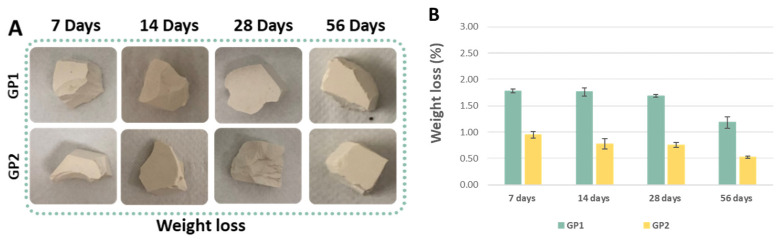
(**A**) GP1 and GP2 images after the weight loss tests. (**B**) GP1 and GP2 weight loss (%) for different ageing times.

Figure 10A reveals a comparison of the synthesised geopolymers before and after the weight loss tests, whilst Figure 10B reports the results of the tests. The histogram shows that during the ageing, both the samples cured at 25 and 40 °C experienced a reduction in the weight loss percentage, suggesting an improvement in the chemical stability over time. Moreover, the geopolymers cured at 40 °C lost less weight than those cured at 25 °C. The little differences between the weight loss of the GP1 and GP2 samples suggested a better geopolymerisation after the heat treatment at 40 °C. Furthermore, the histogram also reveals that all the samples lost less than 2% (GP1) and 1% (GP2) of their initial weights, revealing the high stability of the densified samples.

**Figure 11:**
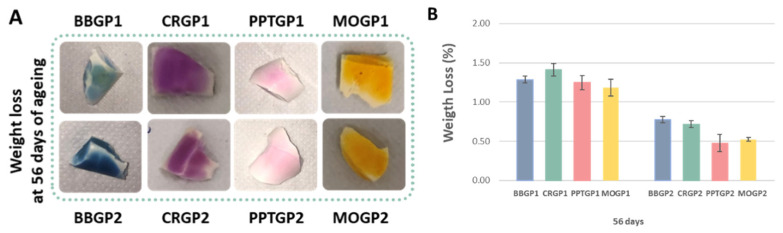
(**A**) Coloured geopolymers images after the weight loss tests. (**B**) Coloured geopolymer weight loss (%) at 56 days of ageing.

The results of the weight loss tests of the coloured geopolymer samples at 56 days of ageing are reported in Figure 11A,B. The weight loss percentages were comparable to those obtained for the white geopolymers cured at 25 or 40 °C and tested after 56 days of ageing time. The results reported in the histograms (Figure 11B) show that the geopolymers were stable and that the heat treatment at 40 °C resulted in a more compact structure with weight losses lower than those of the samples cured at 25 °C.

#### 3.1.3. FT-IR Analysis

**Figure 12:**
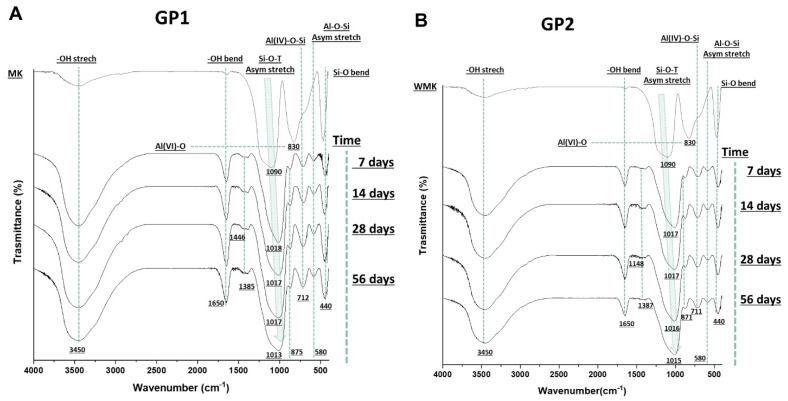
FT-IR spectra of (**A**) GP1 and (**B**) GP2 tested at different ageing times and compared with the MK spectrum.

Fourier transform infrared spectroscopy was used to evaluate the covalent bonds inside the synthesised materials (compared with the metakaolin precursor) and to obtain indirect data about the geopolymerisation occurrence. The density of state peak maximum (DOSPM) [12] was evaluated. Figure 12A,B show the spectra of metakaolin and geopolymer samples (cured at 25 or 40 °C) after sample removal from the moulds at different ageing times. All the spectra possessed the stretching and bending vibrations of -OH at 3450 and 1650 cm^−^^1^ [13,14]. These vibration modes were strong in the geopolymer samples and weak in the MK, which could be explained by the formation of free water during the polycondensation reactions that led to the geopolymeric 3D structures. The bands in the range of 1448–1385 cm^−^^1^ were assigned to the O-C-O vibrations [15] due to the formation of sodium carbonate on the geopolymer surfaces [16]. In addition, these bands were lower in GP2, maybe due to the heat treatment. The peak at 830 cm^−^^1^ in the MK spectrum was assigned to Al(VI)-OH or Al(VI)-O, and it vanished during the geopolymerisation. New bands at 712–711 cm^−^^1^, assigned to the bending vibration of Al(IV)-O-Si, appeared in the geopolymer samples at different curing and ageing times. These bands suggest the transition of Al(VI) (in MK) to Al(IV) after geopolymerisation [17,18]. Moreover, the geopolymer spectra revealed that the bands at 580 and 440 cm^−^^1^ were Si-O-Al stretching and Si-O bending, respectively [14]. Finally, the geopolymerisation occurrences could be indirectly followed by the shift to the lower wavenumber of the asymmetric stretching of Si-O-T (T = Si or Al), known as DOSPM, which located at 1090 cm^−^^1^ for the metakaolin and 1018–1013 cm^−^^1^ for the geopolymer spectra [12,19,20,21].

**Figure 13:**
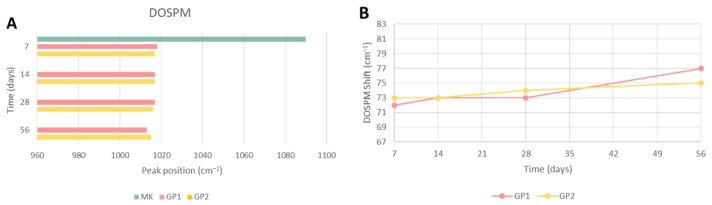
DOSPM of (**A**) MK, GP1, and GP2. (**B**) DOSPM shift from MK recorded at different ageing times.

Figure 13A reports the DOSPM peak position of MK and GPs at different curing and ageing times, whilst Figure 13B reports the respective shifts from the MK DOSPM. The results revealed no differences between the shifts of the samples treated at 25 or 40 °C. FT-IR could not be used to quantitatively evaluate the extent of the 3D-reticulation; yet, it confirmed that the nature of the aluminosilicate network was the same for the two curing temperatures.

**Figure 14:**
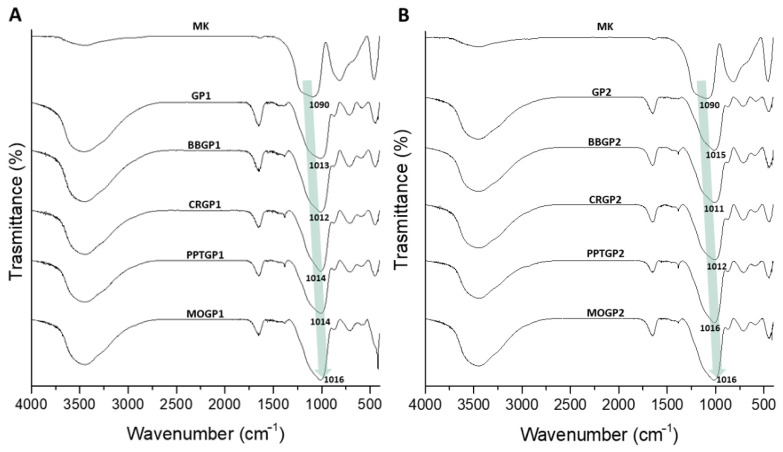
FT-IR spectra of: (**A**) GP1, BBGP1, CRGP1, PPTGP1, and MOGP1; (**B**) GP2, BBGP2, CRGP2, PPTGP2, and MOGP2 extracted at 56 days of ageing times and compared with the MK spectrum.

Figure 14A,B report the spectra of the coloured geopolymers. The spectra show that all the samples had geopolymerised; indeed, there were DOSPMs shifts at lower wavenumbers. Moreover, it can be seen that the DOSPM values were very close to those of GP1 and GP2 after 26 days of ageing, suggesting that the synthetic organic dyes incorporated into the geopolymers did not negatively affect the geopolymerisation. We also observed that the amount of organic dyes was so small as not to be detected by this vibrational spectroscopy. A comparison between the sample spectra with and without the dyes (Figure 12 and Figure 14, respectively) shows that there was a little difference due to a sharp absorption band at 420 cm^−^^1^, visible in the spectra within the dyes.

#### 3.1.4. Leaching Test

**Figure 15:**
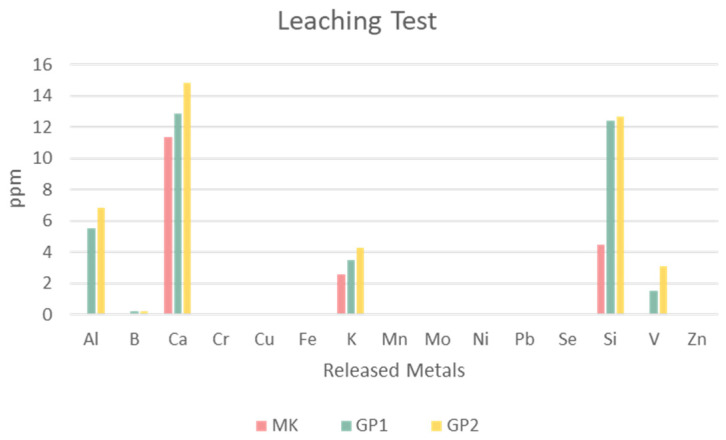
Leaching test of MK powder and GP1 and GP2 extracted after 56 days of ageing. The error due to the preparation procedure is between 20% and 30%.

Figure 15 reports the values in parts per million of the heavy ionic metals released by MK, GP1, and GP2. The histogram reveals that the main elements released were Al, Ca, K, Si, and V, their amounts being proportional to the content, except for silicon. The precursor MK released a low amount of these metals; there was a growing amount of released metals after the geopolymerisation. This could have happened because of alkalinisation. Moreover, the curing temperature also influenced the metal release; the results showed that GP2 released a slightly higher amount of metals than GP1. Leaching of Al and Si was very similar for GP1 and GP2, whilst Ca, and in a minor amount K, increased with alkaline media (passing from MK to geopolymers) as well as with temperature. Leaching of V became detectable in alkaline media, yet remained at the level of impurity.

## 4. Conclusions

The investigated geopolymers formulations showed good compatibility with organic dyes, not significantly changing their microstructural 3D network, as indicated by the vibrational spectroscopy results in the infrared range, as well as the chemical stability, as indicated by the release of unreacted alkaline media influencing the pH and ionic conductivity of the leachate water. Colours did not visibly suffer from curing at 40 °C, except for the case of phenolphthalein, which naturally decomposes at pH higher than 10 to the colourless form. Colours were retained after immersion in water for a short time; hence, the use of these materials for exterior decorations is not suggested.

## Figures and Tables

**Table 1:** Chemical composition of MK.

SiO_2_	Al_2_O_3_	TiO_2_	Fe_2_O_3_	K_2_O	V_2_O_5_	SO_3_	CaO	ZrO_2_	Cr_2_O_3_	ZnO	CuO	PbO	MnO	NiO	MoO_2_
52–54%	37–44%	2.5–7.6%	<0.5%	<0.5%	<0.3%	<0.2%	<0.2%	<0.1%	<0.1%	<0.1%	<0.05%	<0.05%	<0.05%	<0.05%	0.03%

**Table 2:** (**A**) ∆E of the samples (24 h at 25 °C) compared with that of white sample GP1. (**B**) ∆E of the samples (24 h at 40 °C) compared with that of white sample GP2.

A		B	
Sample	∆E	Sample	∆E
GP1	0	GP2	0
MOGP1	44.55	MOGP2	41.31
PPTGP1	47.46	PPTGP2	42.37
CRGP1	49.32	CRGP2	64.71
BBGP1	57.89	BBGP2	56.44

**Table 3:** (**A**) ∆E of the samples (24 h at 25 °C) after 1 h release test compared to white sample GP1 (**B**) ∆E of the samples (24 h at 40 °C) compared to white sample GP2.

A		B	
Sample	∆E	Sample	∆E
GP1	0	GP2	0
MOGP1	48.14	MOGP2	48.27
PPTGP1	70.09	PPTGP2	52.54
CRGP1	67.37	CRGP2	59.30
BBGP1	61.44	BBGP2	57.47

**Table 4:** Summary of the results of the integrity test vs. ageing time.

Samples	Test Parameters	7 Days	14 Days	28 Days	56 Days
GP1	pH	10.2 *	9.00 *	10.5 *	9.8 *
	Resistance	No	Yes	Yes	Yes
	Water	Clear	Clear	Clear	Clear
GP2	pH	10.4 *	9.3 *	10 *	10.1 *
	Resistance	Yes	Yes	Yes	Yes
	Water	Clear	Clear	Clear	Clear

* Sample reproducibility stayed within an error of ±1%.

## Data Availability

Further inquiries can be directed to the corresponding author.
